# Validation of a batch cultivation protocol for fecal microbiota of Kenyan infants

**DOI:** 10.1186/s12866-023-02915-9

**Published:** 2023-07-04

**Authors:** Carole Rachmühl, Christophe Lacroix, Ambra Giorgetti, Nicole U. Stoffel, Michael B. Zimmermann, Gary M. Brittenham, Annelies Geirnaert

**Affiliations:** 1 Laboratory of Food Biotechnology, Institute of Food, Nutrition and Health, Department of Health Sciences and Technology, ETH Zürich, Zurich, Switzerland; 2 Laboratory of Human Nutrition, Institute of Food, Nutrition and Health, Department of Health Sciences and Technology, ETH Zürich, Zurich, Switzerland; 3 Department of Pediatrics, College of Physicians and Surgeons, Columbia University, New York, USA

**Keywords:** In vitro gut fermentation, African infant, pH, Fecal sample preservation, Metabolites

## Abstract

**Background:**

The combination of cultivation studies with molecular analysis approaches allows characterization of the complex human gut microbiota in depth. In vitro cultivation studies of infants living in rural sub-Saharan Africa are scarce. In this study, a batch cultivation protocol for Kenyan infant fecal microbiota was validated.

**Methods:**

Fresh fecal samples were collected from 10 infants living in a rural area of Kenya. Samples were transported under protective conditions and subsequently prepared for inoculation within less than 30 h for batch cultivation. A diet-adapted cultivation medium was used that mimicked the daily intake of human milk and maize porridge in Kenyan infants during weaning. 16 S rRNA gene amplicon sequencing and HPLC analyses were performed to assess the composition and metabolic activity, respectively, of the fecal microbiota after 24 h of batch cultivation.

**Results:**

High abundance of *Bifidobacterium* (53.4 ± 11.1%) and high proportions of acetate (56 ± 11% of total metabolites) and lactate (24 ± 22% of total metabolites) were detected in the Kenyan infant fecal microbiota. After cultivation started at an initial pH 7.6, the fraction of top bacterial genera (≥ 1% abundant) shared between fermentation and fecal samples was high at 97 ± 5%. However, *Escherichia-Shigella*, *Clostridium sensu stricto* 1, *Bacteroides* and *Enterococcus* were enriched concomitant with decreased *Bifidobacterium* abundance. Decreasing the initial pH to 6.9 lead to higher abundance of *Bifidobacterium* after incubation and increased the compositional similarity of fermentation and fecal samples. Despite similar total metabolite production of all fecal microbiota after cultivation, inter-individual differences in metabolite profiles were apparent.

**Conclusions:**

Protected transport and batch cultivation in host and diet adapted conditions allowed regrowth of the top abundant genera and reproduction of the metabolic activity of fresh Kenyan infant fecal microbiota. The validated batch cultivation protocol can be used to study the composition and functional potential of Kenyan infant fecal microbiota in vitro.

**Supplementary Information:**

The online version contains supplementary material available at 10.1186/s12866-023-02915-9.

## Background

The composition and functional potential of the infant gut microbiota is traditionally characterized by longitudinal metagenomic and metabolite analysis of feces [1–4]. Fecal metabolite-based analysis of functional potential, however, is restricted to metabolites that escape host intestinal absorption [5]. Intestinal metabolite absorption is affected by gut transit, permeability and metabolite transportation, which differ between hosts [6, 7]. Cultivating the fecal microbiota allows analysis ex vivo of the microbial metabolite production capacity under controlled conditions. Gut microbiota cultivation allows also study of microbiota mechanisms independent of the host and can be used to investigate whether differences in gut microbiota composition actually translate into functional differences [8, 9].

A major challenge in gut microbiota cultivation is to regrow a representative community of the donor microbiota. Immediate processing of fresh fecal samples is often not feasible due to complex logistics or geographical spread of the sampling and processing sites [10, 11]. Therefore, appropriate handling and preservation of the fecal community and well-chosen cultivation conditions must be selected. A frequently used preservation method for fecal samples is rapid freezing at -20 °C or -80 °C with or without cryoprotectant [12, 13]. Freezing without cryoprotectant, however, was shown to decrease community cultivability and diversity [13]. The stability of propionate-producing *Bacteroidaceae*, an important functional group of the infant gut, was mostly affected after freezing of artificial colonic microbiota with cryoprotectants [14]. The use of fresh feces is recommended for gut microbiota cultivation, which implies limited transportation time and protective conditions [15]. The preservation conditions should prevent microbial growth and minimize the exposure to oxygen to avoid loss of obligate anaerobes [15–17]. Compositional and functional stability of fecal communities were demonstrated after 48 h of cold storage in deoxygenated preservation buffer prior to cultivation [18]. Generation of an anaerobic atmosphere immediately after stool collection ensured viability of oxygen-sensitive bacteria such as *Akkermansia muciniphila*, a prevalent species in the infant gut [16]. Finally, formulation of the cultivation medium to reflect the diet of the host is essential because the in vitro community composition and activity are modulated by the nutrient types and concentrations [19–21].

Many in vitro cultivation studies have been conducted using fecal microbiota of Western infants while fecal microbiota cultivation studies of non-Western infants, including infants living in rural areas of sub-Saharan Africa, are scarce [22]. Fecal batch cultivations were performed with samples of pre-weaning and weaning infants living in a rural location of Malawi to assess their capability to ferment resistant starch [23]. The fecal samples were frozen in liquid nitrogen without protectant prior to cultivation in basal nutritive medium containing tryptone, yeast extract and mineral salts. Fecal samples of undernourished infants living in rural Burkina Faso were cultivated continuously to assess the response to a prebiotic-enhanced lipid-based nutrient supplement [24]. The fecal samples were frozen at -80 °C without protectant prior to cultivation in a standard basic feed with reduced amounts of nutrients to mimic malnutrition status. Freezing of fecal samples without protectant prior to cultivation in a nutritive medium lacking close adjustment to the infant diet can affect cultivability and represent limitations of these previous studies [23, 24]. Further, both studies did not report on the similarity between fermentation and fecal samples, which is needed to assess whether a representative community of the target host fecal microbiota was established after cultivation.

The aim of this study was therefore to assess whether the abundant bacterial genera and metabolic activity of fresh Kenyan infant fecal microbiota can be reproduced after cooled anaerobic transport and batch cultivation in a medium designed to closely mimic the diet of Kenyan infants during weaning [20]. Fresh feces of 5–9 months old Kenyan infants were transported from a remote area in Kenya to Zurich in Switzerland and used for inoculation of fecal batch fermentations within less than 30 h after collection. Microbiota composition and metabolite profiles of fecal and 24 h fermentation samples were compared using 16 S rRNA gene amplicon sequencing and HPLC analysis, respectively.

## Methods

### Fecal donor characteristics

Fresh fecal samples were collected from 10 infants (5 male, 5 female) living in Msambweni County in southern coastal Kenya (Table 1). All infants had not received antibiotics and no illness was reported for the 12 weeks period before sample donation. The age of the infants was 7.0 ± 1.4 months. All infants, except infant 06, were born vaginally. Infant 01 to 06 were born at term while infant 07 to 10 were born at late preterm in week 36. The mean birth weight of the infants was 2.9 ± 0.7 kg. Nine infants were fed with mixed diets of mother milk and complementary foods that consisted predominantly of a maize porridge called “uji” besides vegetables and fruits. Only infant 03 was still exclusively breast-fed. The mean weaning time was 1.4 ± 1.1 months. The majority (80%) of infants were exclusively breast-fed until 6 months, two infants until 5 months and one infant until 4 months. The fecal pH was 5.2 ± 0.9.

**Table 1:** Characteristics of the fecal donors and measured fecal pH

Infant	Age (months)	Feeding	Weaning time (months)	Delivery	Gestation at birth	Birth weight (kg)	Fecal pH
01	7–8	mixed	1.6	Vaginal	Term	3.4	4.2
02	6–7	mixed	0.2	Vaginal	Term	3.7	5.2
03	5–6	mother milk	0.0	Vaginal	Term	2.0	4.7
04	5–6	mixed	1.6	Vaginal	Term	3.5	4.2
05	6–7	mixed	0.1	Vaginal	Term	3.2	5.4
06	5–6	mixed	0.4	Caesarian	Term	2.1	5.8
07	8–9	mixed	2.6	Vaginal	Preterm (36w)	na	4.9
08	8–9	mixed	2.6	Vaginal	Preterm (36w)	na	5.4
09	8–9	mixed	2.4	Vaginal	Preterm (36w)	3.0	4.6
10	8–9	mixed	2.0	Vaginal	Preterm (36w)	2.5	7.3

Mixed: mother milk and complementary foods, na: not available, w: weeks

### Fecal sample collection and transport

**Fig. 1:**
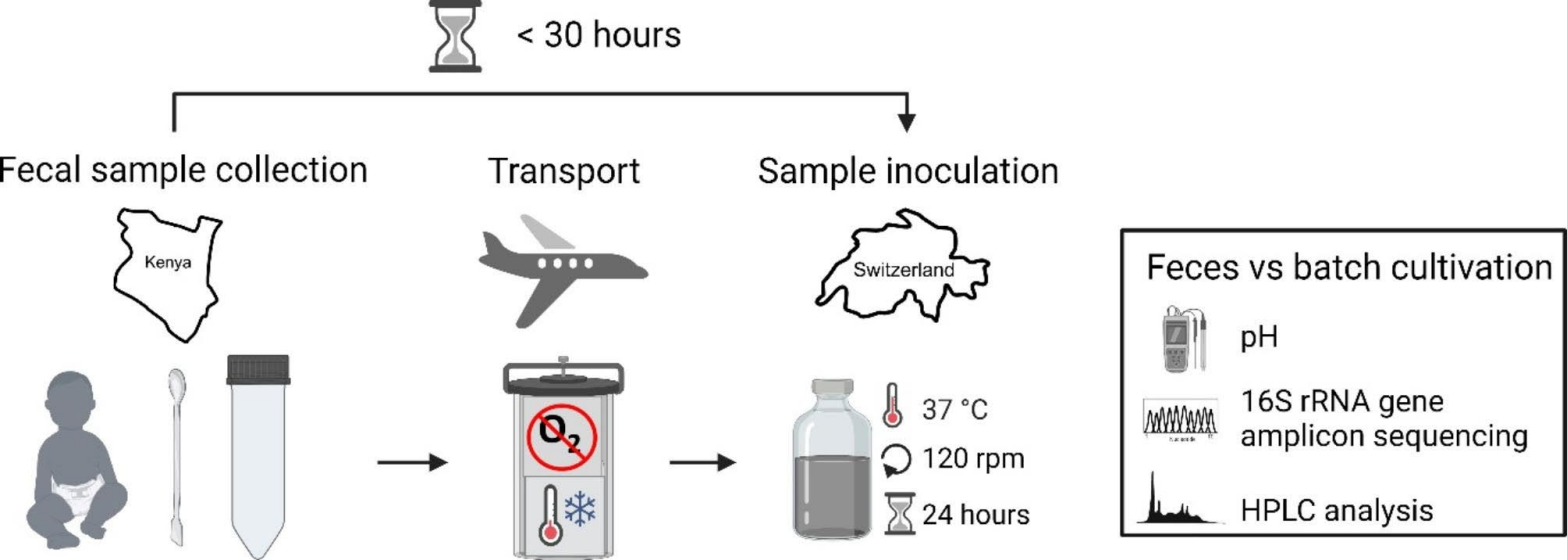
Schematic illustration of fecal sample collection, transport and processing. Created with BioRender.com

An overview of fecal sample collection, transport and processing is illustrated in Fig. 1. The fecal samples were aliquoted from diapers into Falcon tubes and immediately transferred to a gas-tight anaerobic jar containing an anaerobic atmosphere generating system (AnaeroGen, Thermo Fisher Diagnostics AG, Pratteln, Switzerland). For infant 07 to 10 a fecal aliquot was also collected using the DNA-stabilizing OMNIgeneGUT collection kit (DNA Genotek, Ottawa, Canada) to compare the fresh and stabilized fecal community after transport. Samples were cooled with cold packs and air-transported from Kenya to Zurich in two separate batches (01–06; 07–10) for further processing and inoculation within 21 to 29 h after collection. The sample temperature ranged from 4 to 7 °C upon arrival in the laboratory. Fecal sample aliquots were stored at -80 °C until further use for DNA extraction and pH measurement.

### Fecal pH measurement

Fecal pH was measured as previously described [25] with minor modifications. In brief, 100–120 mg of fecal sample was mixed with 1 mL of dH_2_O and homogenized by vortexing.

The pH of the homogenized solution was measured using a pH electrode (Methrom, Zofingen, Switzerland).

### Cultivation medium specific for the Kenyan infant gut microbiota

The cultivation medium was adapted from Doo et al. [20] to mimic the ileal chyme entering the proximal colon of Kenyan infants aged 6 to 8 months after the introduction of complementary foods. The representative daily diet consists of 500 mL mother milk and 330 g uji, a maize or millet porridge. The cultivation medium designed by Doo et al. contained lactose to mimic the milk-derived carbohydrate source and corn starch, arabinogalactan and xylan to mimic plant-derived carbohydrate sources. Further, the medium contained casein and whey protein hydrolysate to mimic the milk-derived protein sources and zein and gluten hydrolysate from maize to mimic the maize-derived protein sources. The medium did not contain additional iron besides hemin to mimic the low iron bioavailability in the infant’s porridge diet. In this study, 1 g/L of fructo-oligosaccharides (FOS, Fibrulose F97, Cosucra, Pecq, Belgium) were added additionally to mimic the presence of FOS in vegetables and fruits that are part of the Kenyan infant complementary food [26, 27]. The detailed composition of the cultivation medium is shown in Supplementary Information. The medium components were dissolved in distilled water. To remove oxygen, the mixture was heated until boiling using a heating plate (IKA, Staufen, Germany) and an Allihn condenser (Lenz Laborglas GmbH, Wertheim, Germany) followed by flushing with CO_2_ for 15 min. The medium was filled into CO_2_-flushed serum flasks which were closed with a butyl rubber and sealed with an aluminium cap. After autoclaving at 121 °C sterile-filtered vitamin and FOS solution were added. The initial pH of the sterilized cultivation medium used for fecal batch fermentations of infant 01 to 06 was 7.6. As an enrichment of taxa, which are favoured by high cultivation pH, was observed after cultivation an initial pH 7.6, the pH of the medium was adjusted to 6.9 for batch cultivations of samples from infant 07 to 10.

### Fecal batch fermentations

Immediately after reception, samples were processed under anaerobic conditions (Coy anaerobic chamber (Coy Laboratory Products, Grass Lake, MI, USA) 10% CO_2_, 5% H_2_ and 85% N_2_). Fecal slurries (20% w/v) were prepared by adding 5 g of feces to a Falcon tube containing 20 mL of anaerobic peptone water (pH 7.0) and two sterile glass beads (5 mm diameter). The samples were vortexed for 5 min for homogenization. The fecal slurry (200 µL) was aseptically inoculated through the butyl stopper in a serum flask containing 20 mL of cultivation medium, with technical duplicates. The closed flasks were incubated for 24 h at 37 °C and shaking at 120 rpm using a shaking incubator (Adolf Kühner AG, Birsfelden, Switzerland). Subsequently, outside of the anaerobic chamber, the flasks were opened for measuring the pH and 2 mL of sample were centrifuged for 10 min at 14’000 rpm and 4 °C. The supernatant was used for metabolite analysis, while the sample pellet was stored at -80 °C until further use for DNA extraction.

### 16 S rRNA gene amplicon sequencing analysis and quantitative PCR

The DNA of fecal and fermentation samples was extracted using the FastDNA SPIN Kit for Soil (MP Biomedicals, Zurich, Switzerland) according to the manufacturer’s instructions. Sequencing was performed in technical duplicate from the same fecal DNA extract.

Paired-end 16 S rRNA gene amplicon sequencing was performed using the Illumina MiSeq platform (Illumina, San Diego, CA, United States) at the Genetic Diversity Center (GDC ETH Zürich, Zurich, Switzerland) as described previously [28]. Library preparation included the amplification of the V4 region of the 16 S rRNA gene with the primers 806R (5′-GGACTACHVGGGTWTCTAAT-3′) and 515 F (5′-GTGCCAGCMGCCGCGGTAA-3′) and barcoding of the amplicons with Nextera Index primers. Sequencing was performed with the MiSeq reagent kit v2 (2 × 250 bp read length). Removal of Illumina adaptors and gene-specific PCR primers from raw reads was done using Atropos [29]. The DADA2-pipeline [30] was used to generate amplicon sequencing variants (ASV). Forward and reverse reads were truncated after 170 nucleotides and 160 nucleotides, respectively. Truncated reads with an expected error rate higher than three for forward and four for reverse reads were removed. After filtering, denoising, error rate learning and ASV inference, reads were merged with a minimum overlap of 40 bp. Chimeric sequences were removed and taxonomy was assigned using the SILVA database (v.132) [31]. Median number of reads in the samples was 23,941 (ranging from 16,292 to 74,118). Total number of reads of the included mock community (10 strain even mix genomic material, ATCC MSA-1000) was 27,506.

Quantitative PCR (qPCR) was performed to determine the total 16 S rRNA gene copies in fresh feces and fermentation samples. The Roche Light Cycler 480 System (Hoffmann-La Roche, Basel, Switzerland) was used as described previously [32]. Each reaction mixture contained 5 µl of SensiFAST SYBR No-ROX mix (Labgene Scientific Instruments, Châtel-Saint-Denis, Switzerland), 0.5 µl of forward and reverse primer (10 µM, Microsynth, Balgach, Switzerland), 3 µl of nuclease free H_2_O and 1 µl of DNA template. A 2-step program was used with 3 min of initial denaturation at 95 °C, 40 cycles of 5 s at 95 °C and 30 s at 60 °C and a final melting curve analysis from 65 to 97 °C at a ramp rate of 0.11 °C/s. Standard curves were generated using tenfold dilutions of linearized plasmid containing the gene of interest. Curves showing an amplification efficiency of 86 and 93% (slope − 3.70 and − 3.49) were used for analysis. Reactions were performed in technical triplicates.

### Microbial metabolite analysis

Short-chain fatty acids (SCFA: acetate, propionate, butyrate and valerate), branched-chain fatty acids (BCFA: isobutyrate and isovalerate) and intermediate fermentation metabolites (succinate, lactate and formate) in feces and fermentation samples were quantified using high-performance liquid chromatography (HPLC). Total metabolites represent the sum of SCFA, BCFA and intermediate metabolite concentrations. Fecal metabolites were extracted by dissolving 200 mg of feces in 600 µL 10 mM H_2_SO_4_ followed by centrifugation at 6000 g and 4 °C for 20 min. The fecal and fermentation sample supernatants were filtered through 0.45 μm nylon membranes (Infochroma AG, Zug, Switzerland) into a glass vial and sealed with crimp-caps. A LaChrom HPLC-System (Merck-Hitachi, Tokyo, Japan) was used with a SecurityGuard Cartridge Carbo-H (4 × 3.0 mm; Phenomenex Inc., Torrance, CA, United States) connected to a Rezex ROA-organic acid H + column (300 × 7.8 mm; Phenomenex Inc., Torrance, CA, United States) and an Accela RI detector (Thermo Fisher Scientific Inc., Waltham, MA, United States). Sample volumes of 20 µL were analyzed with a mobile phase of 10 mM H_2_SO_4_ at a flow rate of 0.6 mL/min and a column temperature of 40 °C. The data were processed using EZChrom software (Agilent, Santa Clara, CA, United States).

### Data visualisation and statistical analysis

Microbiota community analysis as well as principal component analysis (PCA) of metabolites was done in R (version 4.0.4) using the phyloseq [33], vegan [34], stats [35], factoextra [36] and ggplot2 [37] packages. Relative abundances and alpha and beta diversity were calculated on rarefied data. Alpha diversity is a measure of diversity within a community with respect to the number of observed taxa (richness) or abundance distribution of taxa (evenness) [38]. Beta diversity is a measure of similarity or dissimilarity between multiple communities [39]. The binary Jaccard similarity index calculates similarity between communities based on presence/absence of taxa. The weighted Jaccard similarity index additionally considers the abundance of the present taxa. Permutational Analysis of Variance (PERMANOVA) was performed on beta diversity indices and multivariate dispersion test was done to test for variance homoscedasticity (full record is shown in Additional file 2). GraphPad Prism (version 9.1.0) was used for creating graphs and for statistical analysis. The Shapiro-Wilk test was done to test for normal distribution. A parametric unpaired t-test was performed to compare two independent data sets.

## Results

### Composition and metabolite profile of the Kenyan infant donor fecal microbiota

**Fig. 2:**
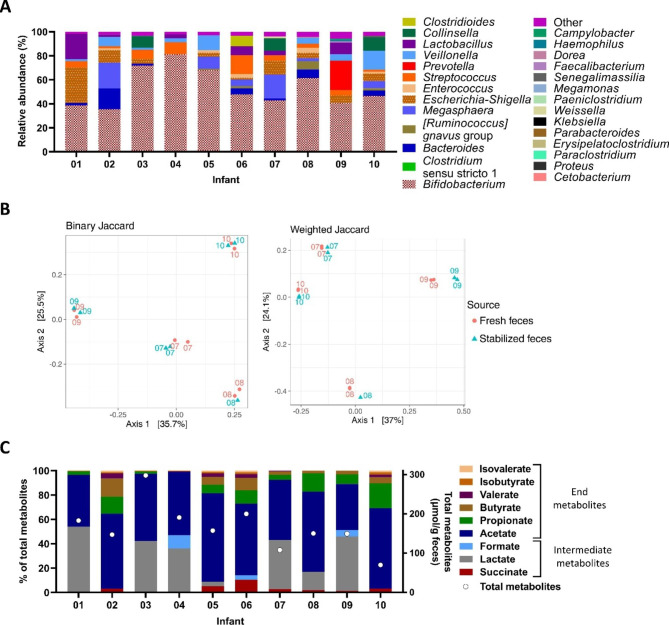
Microbiota composition and metabolite profile of fresh feces based on 16S rRNA gene amplicon sequencing and HPLC analysis. Relative abundance on genus level (“other”: <1%) (**A**). PCoA of binary and weighted Jaccard distance comparing fresh and stabilized feces (**B**). The numbers assigned to symbols denote the infant. Relative proportion (left y-axis) and total absolute concentration (right y-axis, circles) of SCFA, BCFA and intermediate fermentation metabolites (**C**). Averages of technical duplicates are shown except for stabilized feces of infant 08

Microbiota composition and metabolite profile of the fresh feces were determined using 16S rRNA gene amplicon sequencing and HPLC analysis. The most abundant genus in the fresh feces was *Bifidobacterium* (53.4 ± 11.1%), followed by *Escherichia-Shigella* (8.1 ± 4.6%) and *Veillonella* (7.7 ± 6.4%; Fig. 2A). For infant 07 to 10, preservation of the fecal microbiota community structure after transport was assessed by comparing the fresh to DNA-stabilized fecal microbiota. Fresh and stabilized fecal samples of the same infant clustered in the Principal Coordinate Analysis (PCoA) of the beta-diversity metrics binary and weighted Jaccard (Fig. 2B). In accordance, the fecal microbiota composition did not differ between source (fresh or stabilized), but differed significantly between the infants (PERMANOVA, p = 0.000999 for binary and weighted Jaccard). A high fraction (99 ± 3%) of the top bacterial genera (≥ 1%) present in the stabilized fecal samples was detected in the fresh fecal samples, but at different relative abundance compared to the stabilized feces (Additional file 3). Largest differences in abundance were observed between fresh and stabilized fecal samples of infant 08, 09 and 10 for *Bifidobacterium* (difference of 28.7%, 12.3% and 13.7% for infant 08, 09 and 10, respectively), *Prevotella* (difference of 14.3% for infant 09) and *Bacteroides* (difference of 16.2% and 9.9% for infant 08 and 10, respectively).

Total metabolites of the fresh fecal samples differed between the infants (Fig. 2C). The absolute concentration was high at 298 µmol/g feces in infant 03 and low at 70 µmol/g feces in infant 10. The most abundant intermediate metabolite was lactate (24 ± 22% of total metabolites), followed by succinate (3 ± 3%) and formate (2 ± 4%). The most abundant SCFA was acetate (56 ± 11% of total metabolites), followed by propionate (8 ± 7%) and butyrate (4 ± 5%). Inter-individual variability in metabolite proportion was high for all metabolites, except of acetate. The proportions of valerate and BCFA were low in the fecal samples of all infants (ranging from 0 to 4% of total metabolites).

Altogether, the data show that the fecal microbiota of the study population was characterized by dominance of *Bifidobacterium* and high proportions of acetate. The fresh fecal microbiota were most similar to the corresponding stabilized fecal microbiota, despite differences in abundance of the top bacterial genera.

### Regrowth of the top abundant genera of the Kenyan infant fecal microbiota after batch cultivation

**Fig. 3:**
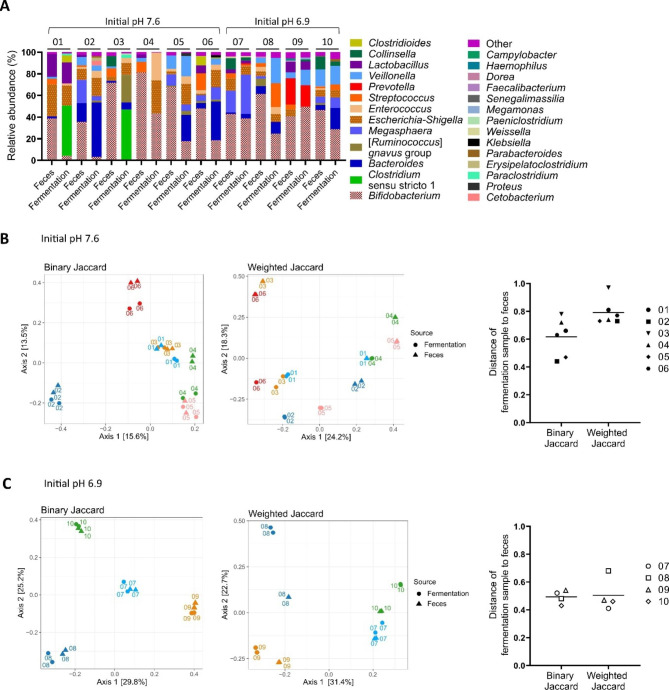
Microbiota composition of fresh feces and after 24 h of fecal batch fermentation based on 16S rRNA gene amplicon sequencing data. Relative abundance on genus level (“other” <1%) (**A**). PCoA and total distance of binary and weighted Jaccard comparing fecal and fermentation samples of infant 01 to 06 (**B**) and 07 to 10 (**C**). Distance of 0 indicates identical communities and distance of 1 indicates different communities. The initial pH of the batch cultivation medium is indicated. The numbers assigned to bars and symbols denote the infant. Averages of technical duplicates are shown for the relative abundance and total distance data

The fresh fecal microbiota were cultivated for 24 h in host-diet-adapted conditions and the final composition was compared to the fecal microbiota. First batch cultivations were performed with feces from infant 01 to 06. The initial pH of the cultivation medium was 7.6 while the final pH ranged from 6.2 to 6.4 (Additional file 4).The fraction of fecal bacterial genera detected in the fermentation samples was 45 ± 12% (Fig. 3A). When the most abundant genera (≥ 1% abundant) were considered, a higher fraction (97 ± 5%) of fecal bacterial genera was detected in the fermentation samples, but at different relative abundances compared to feces. For example, *Bifidobacterium* abundance was lower in all fermentation samples compared to the corresponding fecal samples. Concomitant, *Enterococcus* (infant 01 to 06), *Escherichia-Shigella* (infant 02 to 06), *Bacteroides* (infant 02, 03, 05 and 06), *Clostridium sensu stricto* 1 (infant 01 and 03) and *Ruminococcus gnavus* group (infant 03) were higher in abundance after fermentation compared to the corresponding fecal samples. Fermentation and fecal samples of the same infant clustered in the PCoA of binary Jaccard distance except for infant 04 (Fig. 3B). However, when the relative abundances were considered by using weighted Jaccard, fermentation and fecal samples of the same infant separated in the PCoA. For all infants, the overall composition differed between fermentation and fecal sample based on the beta diversity metrics binary and weighted Jaccard similarity index as indicated by distances higher than 0.4 (Fig. 3B). For fermentation samples of infant 01, 02 and 06, the binary and weighted Jaccard distance was lower compared to the corresponding fecal sample than compared the other infant fecal samples (Additional file 5: Supplemental Fig. 5A).

The distribution among taxa abundance was more even after cultivation compared to the fecal samples as indicated by a significant higher Pielou’s index in fermentation compared to fecal samples (p = 0.0111, Additional file 6).

The initial pH of the medium was lowered to 6.9 for the next cultivation experiment, as an enrichment in taxa, e.g. *Bacteroides*, which are favoured by high cultivation pH [40], was observed after cultivation at initial pH 7.6. For the fecal samples that were cultivated at lower initial pH 6.9 (infant 07 to 10), the pH in the fermentation samples after 24 h ranged from 5.7 to 6.0 (Additional file 4).

As observed for infant 01 to 06, a more than 100-fold increase in 16 SrRNA gene copies was detected when comparing fermentation samples after inoculation (log_10_ 7.2 to 8.2 16 S rRNA gene copies/mL) and after cultivation (log_10_ 10.2 to 10.4 16 SrRNA gene copies/mL) (Additional file 7).

Maintenance of *Bifidobacterium* improved after cultivation at initial pH 6.9 compared to 7.6 as indicated by lower decrease in abundance comparing fermentation to fecal samples (Fig. 3A). Also, the fraction of fecal bacterial genera detected in the corresponding fermentation sample was 58 ± 9% and higher after cultivation at pH 6.9 compared to 7.6. Overall, a high fraction (98 ± 5%) of the top fecal bacterial genera (≥ 1% abundant) was detected in the corresponding fermentation samples but at different relative abundances compared to the fecal inoculum (Fig. 3A). *Bifidobacterium* abundance was lower in the fermentation sample of infant 07, 08 and 10 compared to the corresponding fecal sample. Concomitant, *Bacteroides* (infant 07, 08 and 10), *Megasphaera* (infant 07 and 10), *Escherichia-Shigella* (infant 08), *Enterococcus* (infant 08 and 10), *Streptococcus* (infant 08 and 10) and *Veillonella* (infant 08 and 10) were higher in abundance in the fermentation compared to the corresponding fecal sample. In the fermentation sample of infant 09, *Bifidobacterium* and *Veillonella* abundance were higher compared to the fecal sample. Fermentation and fecal samples of the same infant clustered in the PCoA of binary and weighted Jaccard distance except for infant 08, where the fermentation sample separated from the fecal sample based on axis 2 in the PCoA of weighted Jaccard (Fig. 3C). For all infants, the overall composition differed between fermentation and fecal samples based on binary and weighted Jaccard similarity index as indicated by a distance higher than 0.4 (Fig. 3C). However, for all infant samples cultivated at pH 6.9, the binary and weighted Jaccard distance between fermentation and corresponding fecal sample was lower than the distance between fermentation and the other infant fecal samples (Additional file 5). Further, at initial cultivation pH 6.9, the average weighted Jaccard distance of fermentation to fecal samples (infant 07 to 10, 0.50 ± 0.12) was lower compared to the average weighted Jaccard distance observed at initial cultivation pH 7.6 (infant 01 to 06, 0.79 ± 0.09; p = 0.0095; Fig. 3B).

In conclusion, the top abundant genera of the Kenyan infant fecal microbiota regrew after protected transport and 24 h batch cultivation but reached different relative abundance in fermentation compared to fecal samples. A reduced shift between fermentation and fecal samples was observed for infant fecal samples cultivated at initial pH 6.9 compared to the fecal samples cultivated at initial pH 7.6.

### Inter-individual differences in metabolite profiles after batch cultivation

**Fig. 4:**
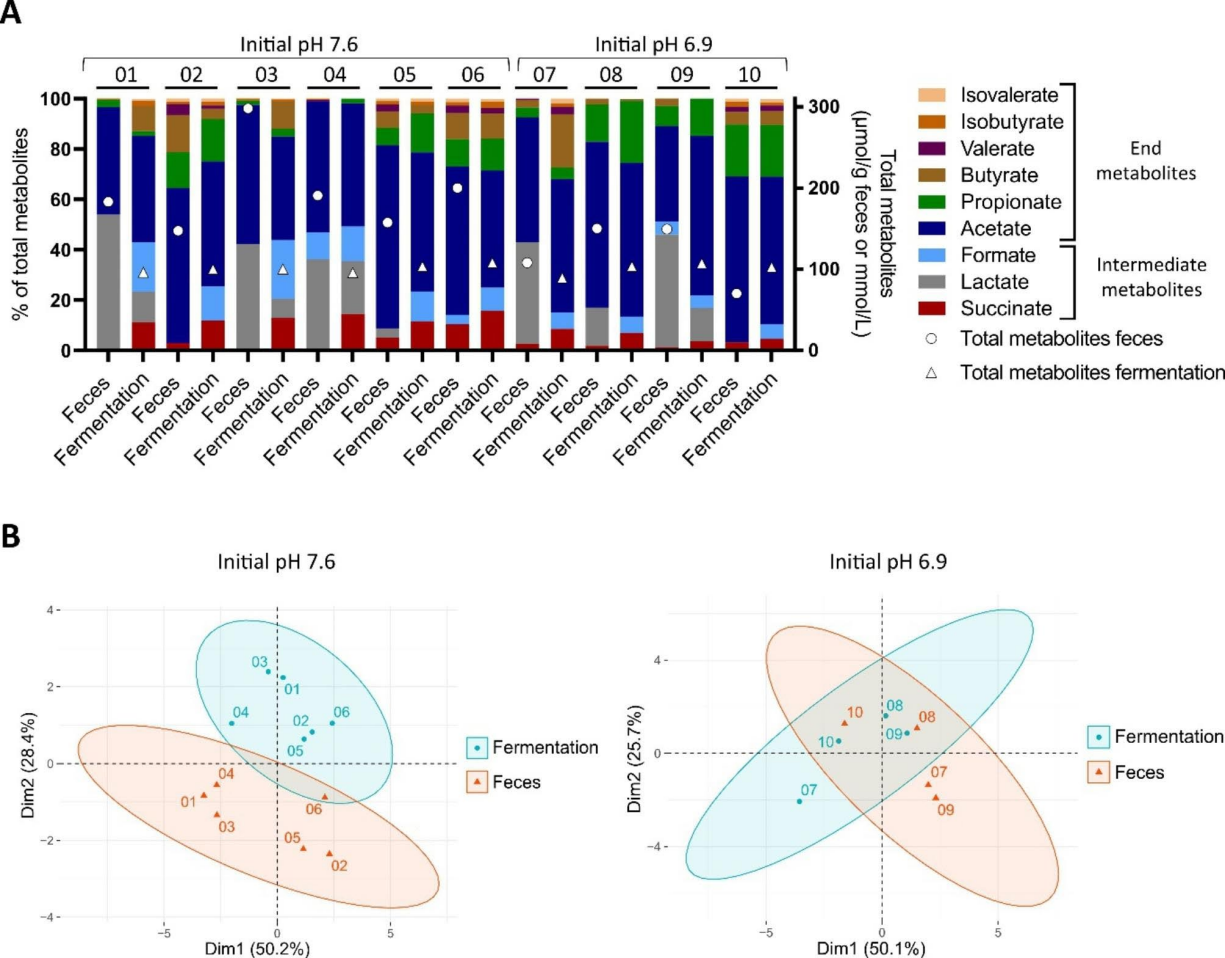
Metabolite profile of fresh feces and after 24 h fecal batch fermentation measured by HPLC. Relative proportion (left y-axis) and total absolute concentration (right y-axis, circles and triangles) of SCFA, BCFA and intermediate metabolites (**A**). PCA of the proportion of metabolites (**B**). The initial pH of the batch cultivation medium is indicated. The numbers assigned to bars and symbols denote the infant microbiota. Averages of technical duplicates are shown

The metabolite profile was analysed after 24 h fecal batch cultivation using HPLC analysis and compared to the metabolite profile of the fecal microbiota. In contrast to the feces, the total metabolite concentration after cultivation was similar for all infant samples (101 ± 6 mM; Fig. 4A). Intermediate fermentation metabolites were detected in all fermentation samples. The most abundant intermediate metabolite for infant 01 to 06 was formate (15 ± 5% of total metabolites), followed by succinate and lactate, while formate (6 ± 1%) and succinate (6 ± 1%) were most abundant for infant 07 to 10, followed by lactate.

Lower total metabolite concentrations and higher proportions of formate and succinate and lower proportions of lactate were generally detected in the fermentation compared to the fecal samples. In the fermentation samples, acetate was the most abundant end metabolite (47 ± 5% and 59 ± 4% of total metabolites for infant 01 to 06 and 07 to 10, respectively), followed by propionate and butyrate. Butyrate was detected in fermentation but not in fecal samples of infant 01 and 03 while propionate was detected in fermentation but not in fecal sample of infant 04.

The proportions of valerate and BCFA were low in fermentation samples of all infants (0–4% of total metabolites). PCA of the metabolite profiles revealed separation of fecal and fermentation samples for infant 01 to 06 and clustering of fecal and fermentation samples for infant 07 to 10 (Fig. 4B). Inter-individual differences in metabolite profile were apparent among the fecal as well as among the fermentation samples.

Altogether, the metabolic activity of the Kenyan infant fecal microbiota was reproduced after protected transport and 24 h batch cultivation. Despite similar total metabolite production by all infant microbiota after cultivation, inter-individual differences in metabolite profiles were observed and profiles were more similar to the fecal metabolite profile for the initial cultivation pH of 6.9.

## Discussion

Cultivation-based methods are used to study the gut microbiota function independent of the host [41]. However, the validity of in vitro gut fermentation is dependent on the selected biotic and abiotic conditions, and on the preservation of the fecal inoculum community and activity. In this study, a protocol for preservation and batch cultivation of fresh Kenyan infant fecal microbiota was validated.

Fecal samples were collected from Kenyan infants aged 7.0 ± 1.4 months of which 90% were already introduced to complementary feeding and 80% were exclusively breast-fed until 6 months of age. Results obtained by several studies conducted at the same study site confirm the representativity of our study population. Introduction to complementary feeding was previously reported in 80.2% of 6-months-old Kenyan infants [42] and exclusive breast-feeding until 6 months was previously reported in 74.6% of Kenyan infants aged 7.7 ± 0.8 months [43]. A high *Bifidobacterium* abundance of 53.4 ± 11.1% was detected in the fecal microbiota of this study. In two previous studies carried out with the same infant population, high *Bifidobacterium* abundance of 62.7% [42] and *Bifidobacteriaceae* (the family harbouring the genus *Bifidobacterium*) abundance of 60.5% [25] were detected in the feces of Kenyan infants aged 6 and 6.5–9.5 months, respectively. The observed high proportions of fecal acetate (56%) and lactate (24%) are consistent with data from a previous study carried out with 28 Nigerian infants during pre-weaning and weaning, reporting mean lactate ratios of 30% that remained high until up to 10 months of age in some infants [44]. However, the fecal pH of 5.2 ± 0.9 measured in this study after refrigerated transport was slightly lower compared to two previous reports with median fecal pH of 5.5–5.7 [25] and an average fecal pH of 5.4 ± 0.8 [45] measured in feces of the same infant population.

The viability and activity of the Kenyan infant fecal microbiota was well preserved during transport as indicated by regrowth of the top genera and reproduction of the metabolic activity after batch cultivation. However, *Escherichia-Shigella*, *Bacteroides*, *Enterococcus* and *Clostridium sensu stricto* 1 were enriched after cultivation at an initial pH 7.6 compared to the fecal inoculum composition. Higher *Bifidobacterium* abundance and similarity of the cultured versus the fecal inoculum microbiota were observed after cultivation at a lower initial pH 6.9. Compositional differences between the cultured and fecal microbiota community are expected because the fecal sample includes microbes as well as biotic and abiotic factors from various parts of the gut and therefore only represents a proxy of the colon microbiota [46]. However, the dominance of *Bifidobacterium* in the feces of the study population suggests that high abundance after fecal batch cultivation is representative of the Kenyan infant gut microbiota [25, 42]. Growth of specific taxa can be favoured during batch cultivation by the ability to grow fast (e.g. for *Escherichia coli*), or by the selection through available nutrients or by cultivation pH [40, 47]. Dominance of *Enterobacteriaceae* and *Enterococcus* was observed after batch cultivation of fecal samples from Malawian and Western infants concomitant with low *Bifidobacterium* abundance [23, 48, 49]. *Bifidobacterium* is among the most abundant in the infant fecal microbiota during the first year of life [2, 50]. The prior cultivation studies did not assess the similarity of fermentation and fecal samples to conclude whether the low *Bifidobacterium* abundances were representative of the infant fecal microbiota or not [23, 48, 49]. Further, feces preservation by freezing prior to cultivation in basal nutritive medium lacking adjustment to closely mimic the infant diet likely affected the growth of the fecal microbiota in vitro. In contrast to the previous studies, our adjusted protocol for preservation and cultivation of fresh African infant fecal microbiota was successful in obtaining high abundance of *Bifidobacterium* and regrowing the top abundant genera after 24 h fermentation in host-diet adapted medium at initial pH 6.9.

The in vitro metabolic activity in our study was higher in comparison to fecal batch cultivations of Malawian and Western infants, which is likely due to different nutrient content of the cultivation medium and optimized protocol used in this study, respectively [23, 48, 51]. Total metabolite levels were similar between the different infant microbiota after fecal batch cultivation, suggesting similar conversion efficiency of available nutrients in the applied cultivation medium. Further, similar total metabolic activity at different initial pH suggests that the batch fermentation was rather nutrient than pH limited. Despite similar total metabolic activity, inter-individual differences in metabolite profiles were apparent after cultivation. In vitro cultivation of fecal microbiota circumvents confounding factors such as nutrient intake and metabolite absorption by the colon epithelium, which affect metabolite levels in feces [5], and therefore allows study of inter-individual differences in metabolite production capacity. The most abundant fermentation end metabolite after cultivation of the Kenyan infant fecal microbiota was acetate followed by propionate and butyrate, as previously reported for fecal microbiota from Malawian and Western infants [23, 48, 52]. High lactate production during cultivation of the Kenyan infant fecal microbiota is expected due to the high abundance of main lactate-producing bacteria, such as *Bifidobacterium, Lactobacillus* and *Bacteroides* [53]. Absence of lactate accumulation observed during batch cultivation of 6 out of 10 Kenyan infant fecal microbiota suggests complete intermediate metabolite utilization and a fully functional cross-feeding network. In particular, the preservation of strict anaerobe lactate-utilizing bacteria under the applied conditions was confirmed, such as for propionate-producing *Veillonella* [53, 54].

## Conclusions

We demonstrated that the top abundant genera and metabolic activity of fresh Kenyan infant fecal microbiota can be reproduced after protected transport and batch cultivation in host-diet-adapted conditions within less than 30 h after collection. This validated batch cultivation protocol is useful to study in vitro the gut microbiota growth and functional potential of infants living in rural sub-Saharan Africa in response to biotic or abiotic factors. Although they are easier to perform than continuous intestinal cultures, batch cultivations do not allow for constant nutrient supply, stable maintenance of pH and elimination of toxic microbial products over time [55]. In a next step, more advanced intestinal continuous fermentation models will allow more closely mimicking the proximal colon conditions [56] and characterization of the dynamics of African infant gut microbiota in vitro.

## Electronic supplementary material

Below is the link to the electronic supplementary material.

Supplementary Material 1

Supplementary Material 2

Supplementary Material 3

Supplementary Material 4

Supplementary Material 5

Supplementary Material 6

Supplementary Material 7

Supplementary Material 8

## Data Availability

The datasets generated and/or analysed during the current study are available in the European Nucleotide Archive (ENA) repository, accession number PRJEB59472 (https://www.ebi.ac.uk/ena/browser/view/PRJEB59472).

## List of Abbreviations

FOS: Fructo-oligosaccharides
ASV: Amplicon sequencing variant
SCFA: Short-chain fatty acids
BCFA: Branched-chain fatty acids
HPLC: High performance liquid chromatography
PERMANOVA: Permutational analysis of variance
PCoA: Principal coordinate analysis
PCA: Principal component analysis
