# Supplementary material for: Validation of a batch cultivation protocol for fecal microbiota of Kenyan infants
Source: BMC Microbiol. 2023 Jul 4;23:174. doi: 10.1186/s12866-023-02915-9 (PMC10318780; doi:10.1186/s12866-023-02915-9)
Supplement: Supplementary file 1 — Supplementary Material 1 [file 12866_2023_2915_MOESM1_ESM.pdf]

**Component**	**g/L**
Zein (47% of total protein)	0.3
Gluten hydrolysate from maize (39% of total protein)	0.3
Corn starch	0.3
Xylan (beechwood)	0.4
Arabinogalactan (larch wood)	2.2
Fructo-oligosaccharides (short-chain)	1.0
D-lactose	3.2
Casein hydrolysate	0.3
Whey protein hydrolysate	4.1
Peptone from casein	0.5
Bacto™ Tryptone	0.5
Mucin	4.0
Yeast extract (Standard nucleotide yeast extract)	2.5
L-cysteine HCl monohydrate	0.8
Bile salts	0.05
KH_2PO_4	3.0
$NaHCO_3$	9.0
NaCl	4.5
KCl	4.5
$MgSO_4$ Anhydrous	1.3
$CaCl_2 \cdot 2H_2O$	0.1
$FeSO_4 \cdot 7H_2O$	0.0
Hemin	0.01
Tween 80	1.0 mL
Vitamin solution	0.5 mL
The composition of the vitamin solution was (mg/L of distilled water):<br>thiamine-HCl (Vit B1-HCl;50), (-)-riboflavin (Vit B2; 50), nicotinic acid (Vit B3; 50), pantotenate (Vit B5; 100), pyridoxine-HCl (Vit B6; 100), folic acid (Vit B9, 20), cyanocobalamin (Vit B12; 5), 4-aminobenzoic acid (PABA; 50),<br>biotine (Vit H; 20), phylloquinone (Vit K1; 0.075) and menadione (Vit K3; 10).	
