# Supplementary figures and images for: Validation of a batch cultivation protocol for fecal microbiota of Kenyan infants
Source: BMC Microbiol. 2023 Jul 4;23:174. doi: 10.1186/s12866-023-02915-9 (PMC10318780; doi:10.1186/s12866-023-02915-9)

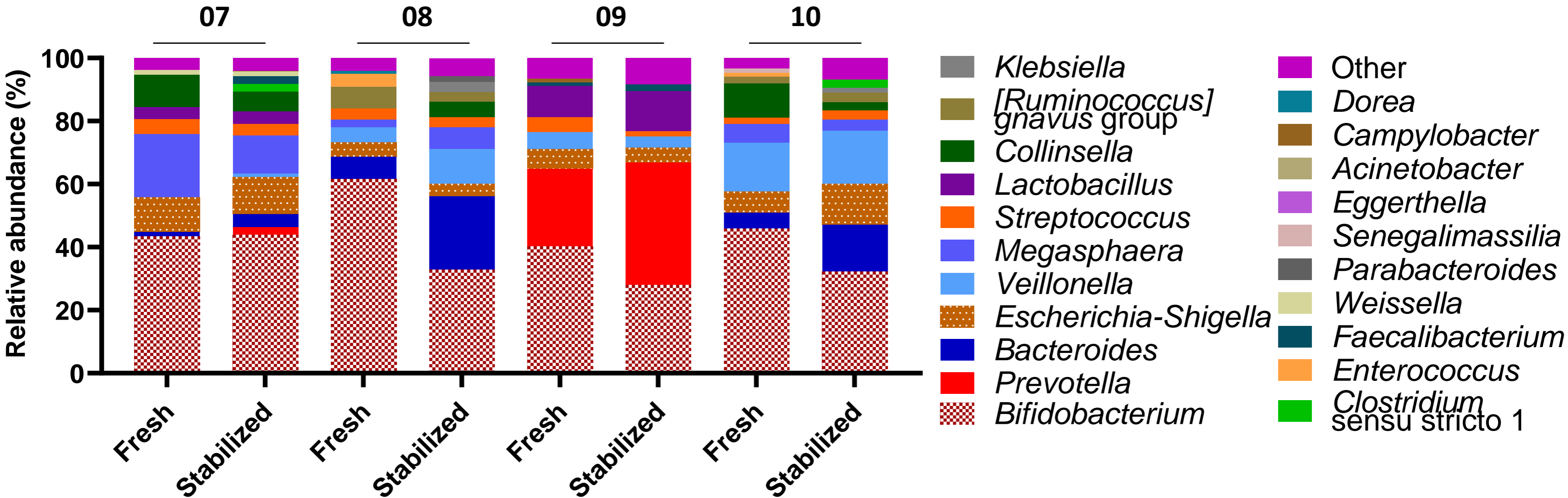

Supplement: Supplementary file 3 — Supplementary Material 3 [file 12866_2023_2915_MOESM3_ESM.pdf]

**A**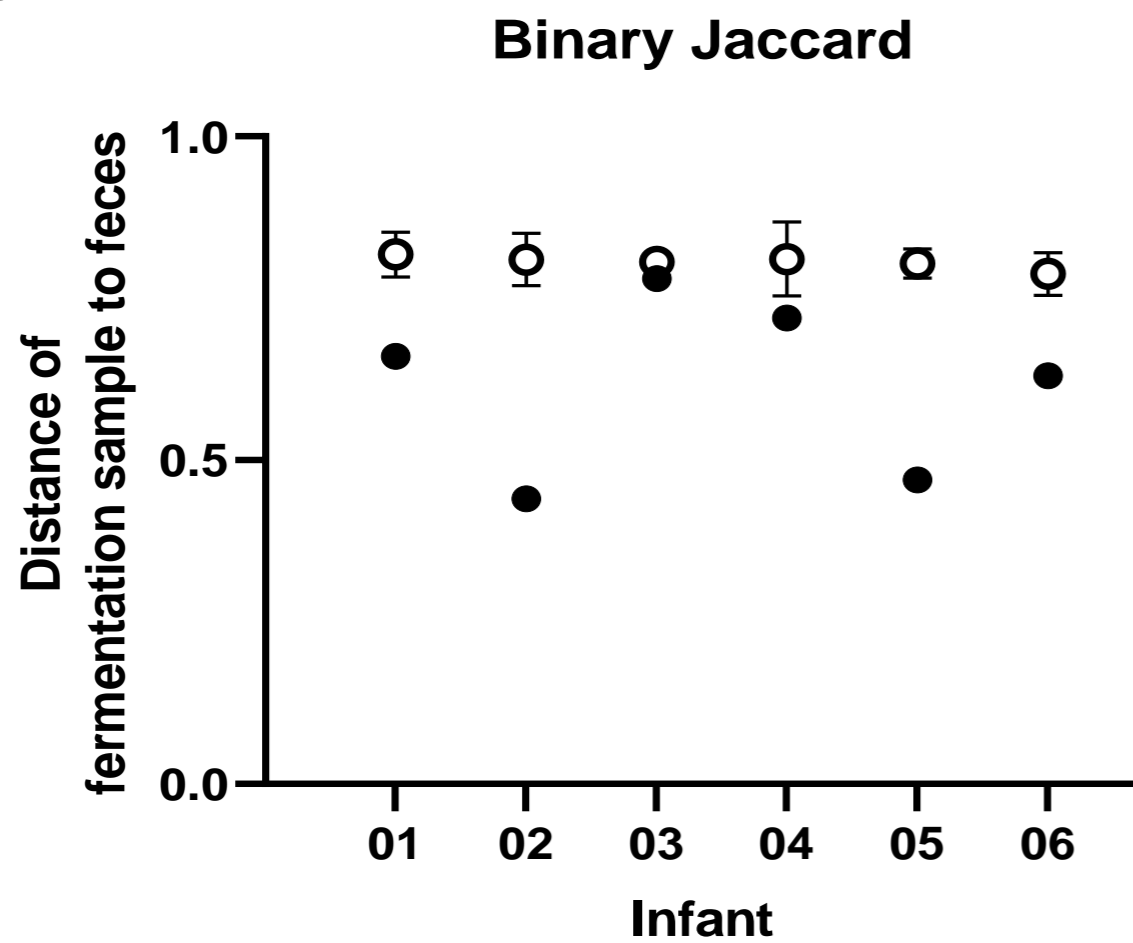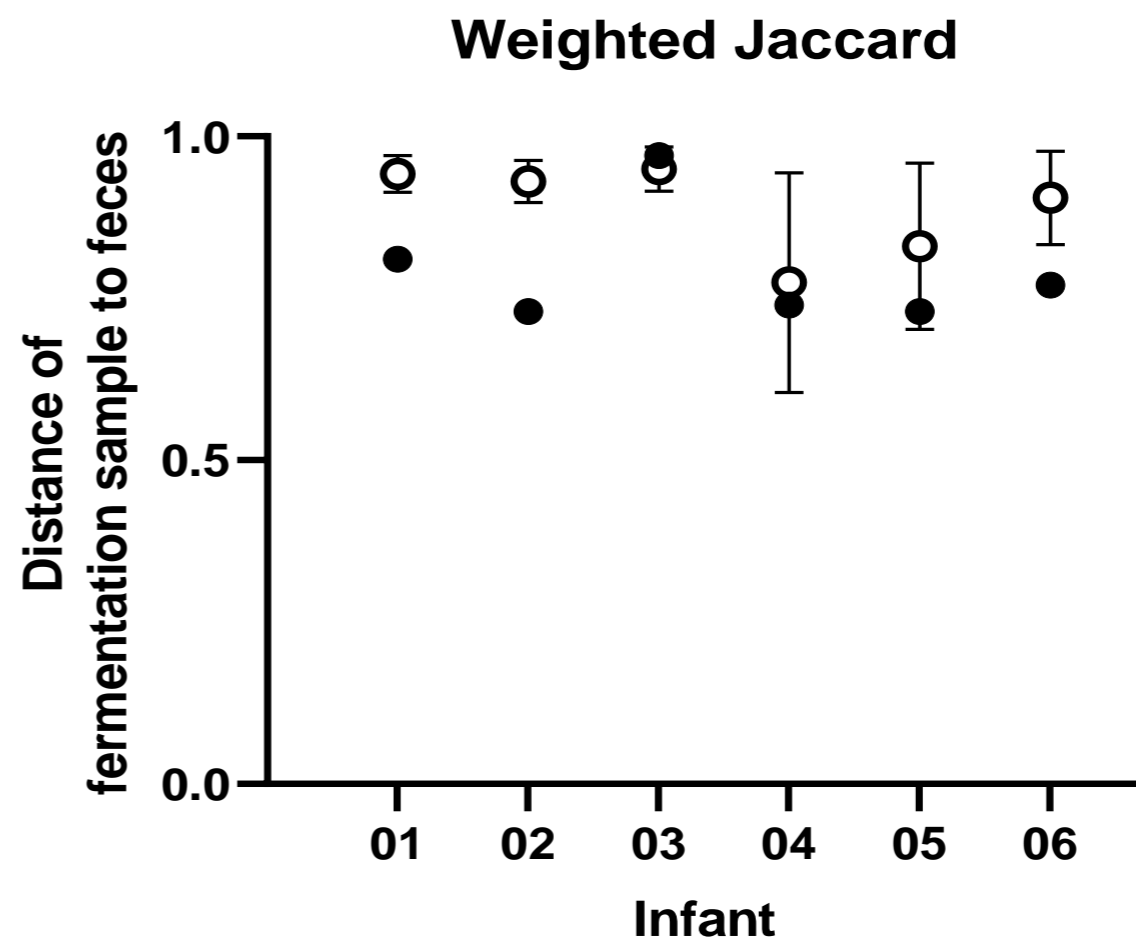

○ other infants  
● within infant

**B**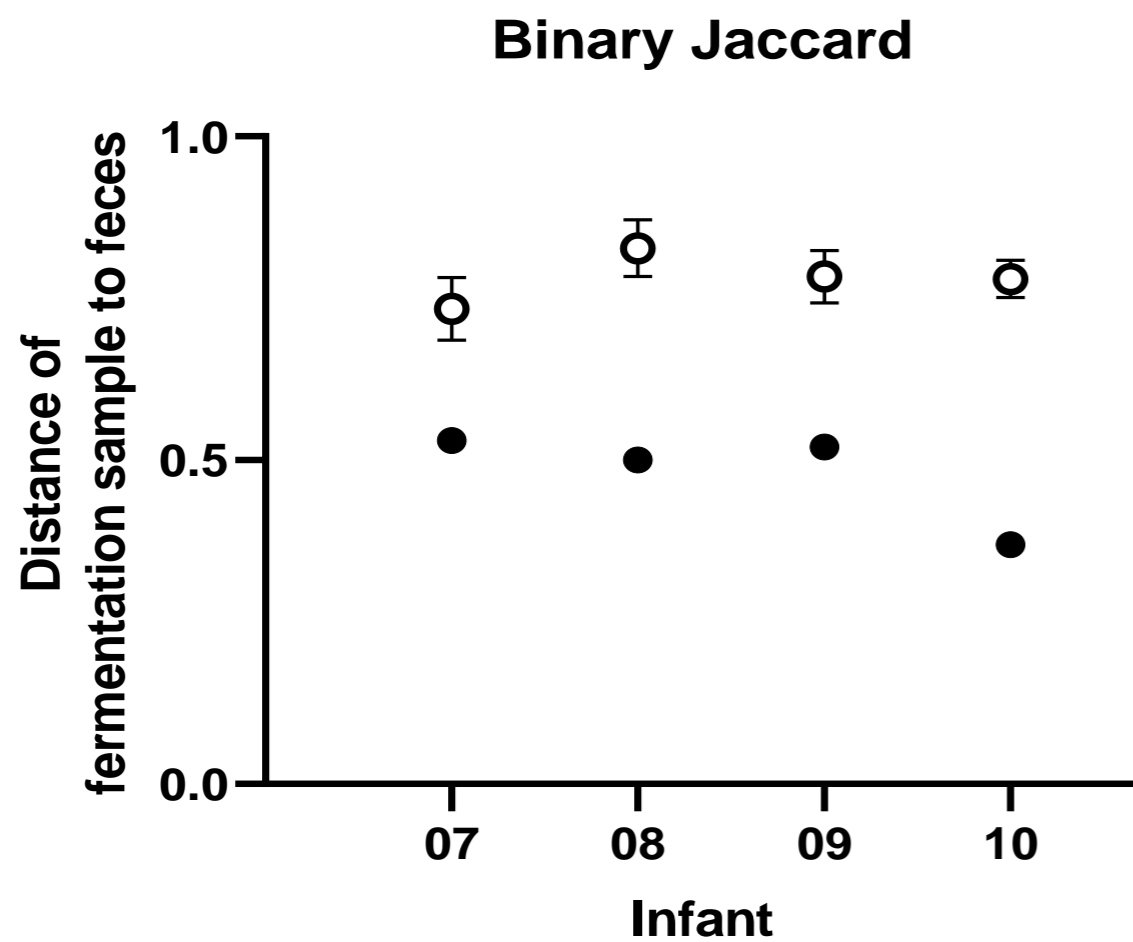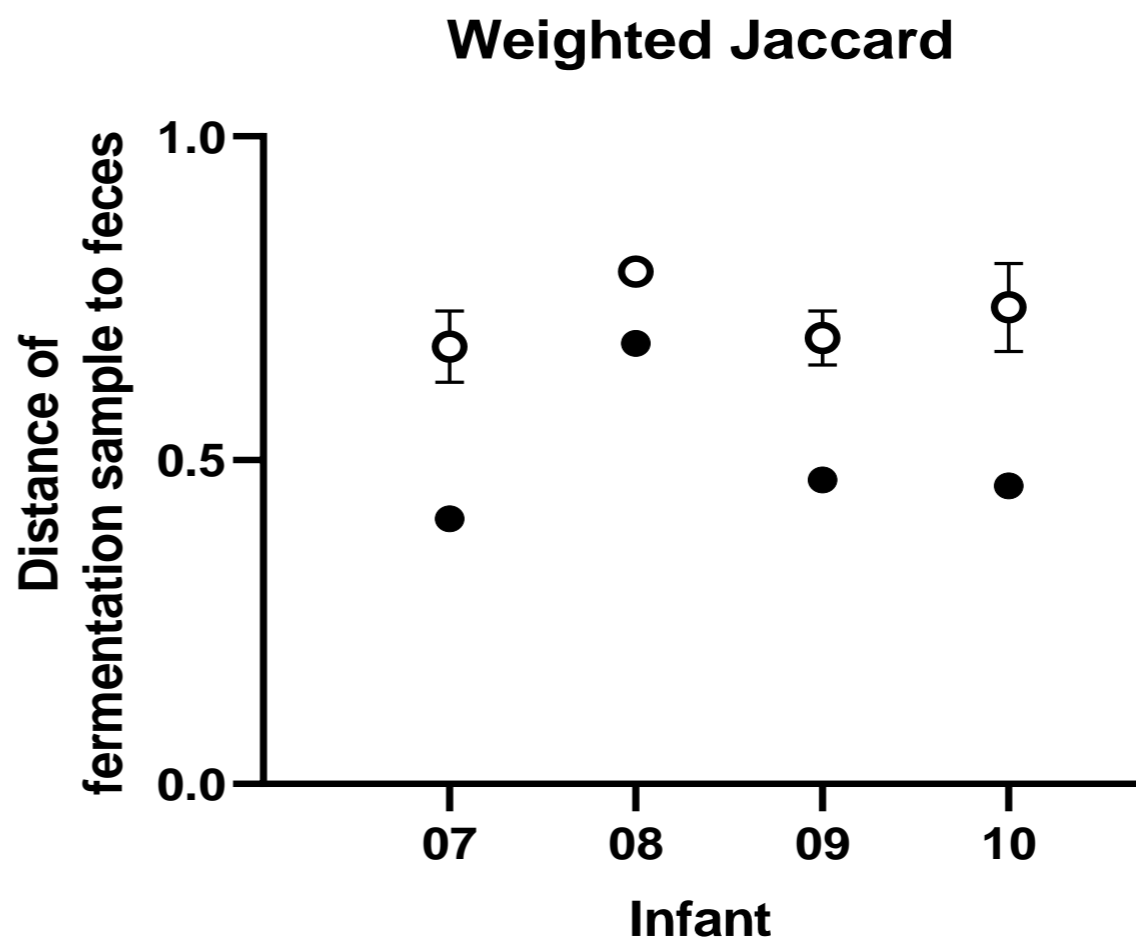

○ other infants  
● within infant

Supplement: Supplementary file 5 — Supplementary Material 5 [file 12866_2023_2915_MOESM5_ESM.pdf]

**A**

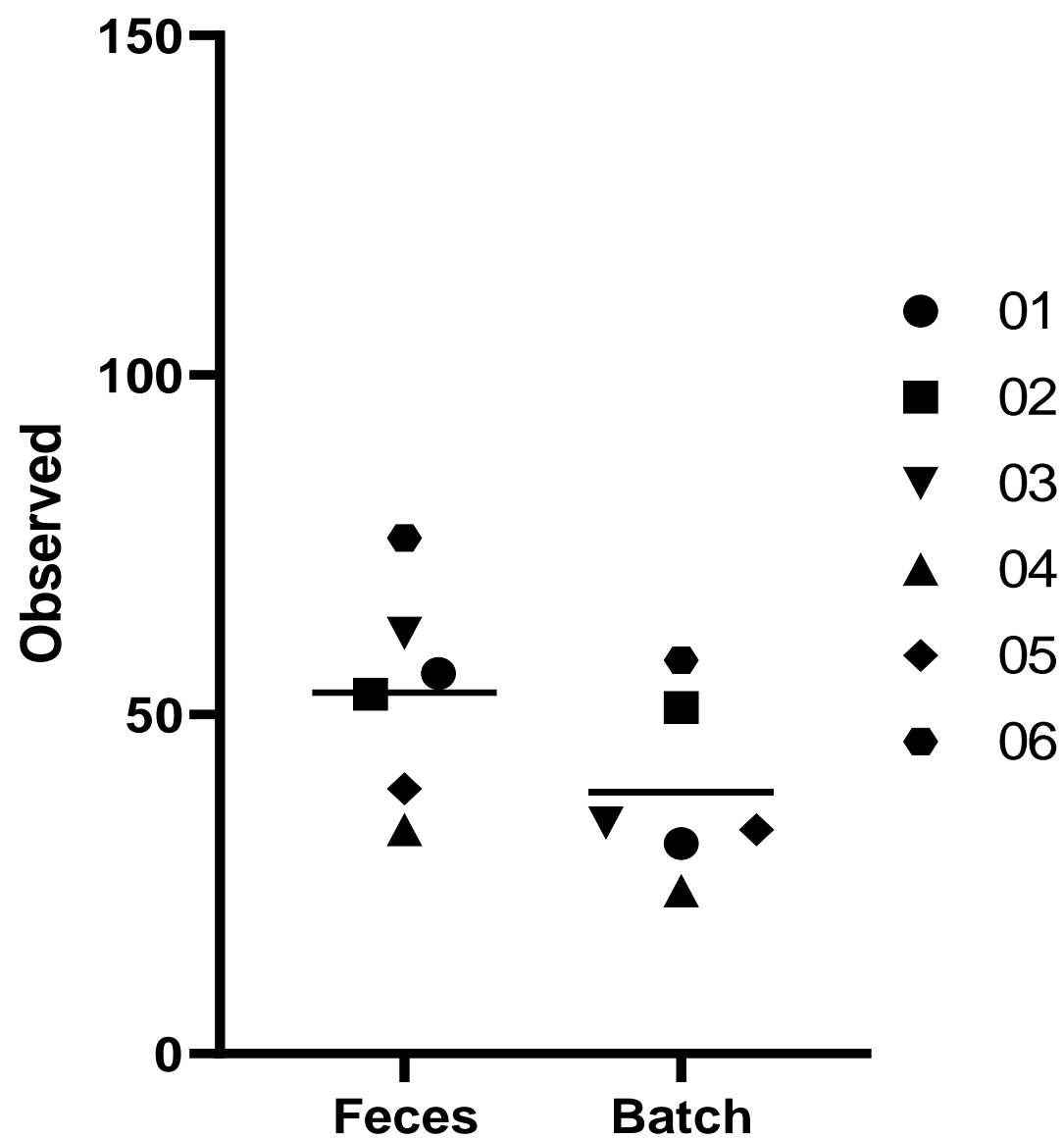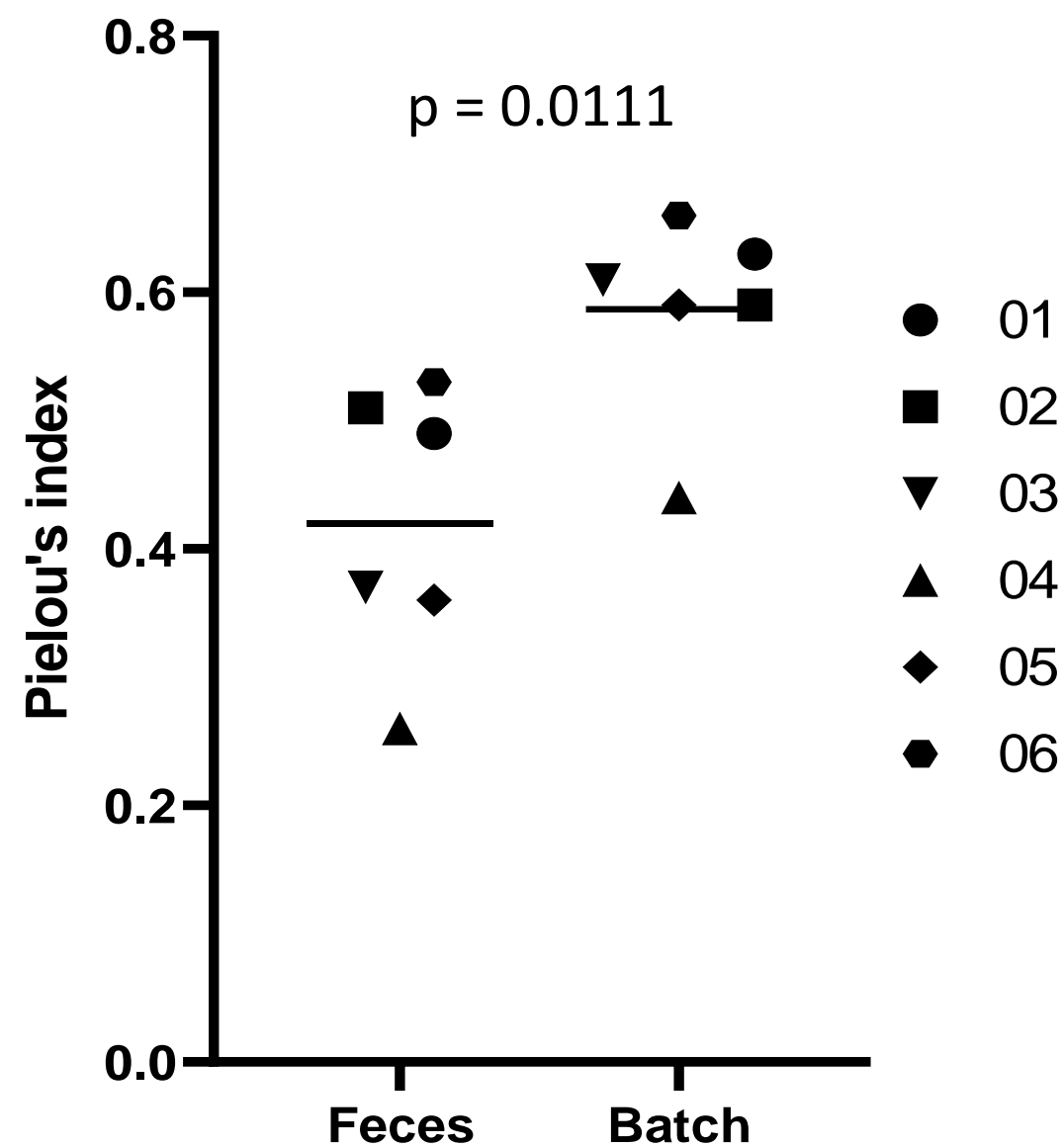

**B**

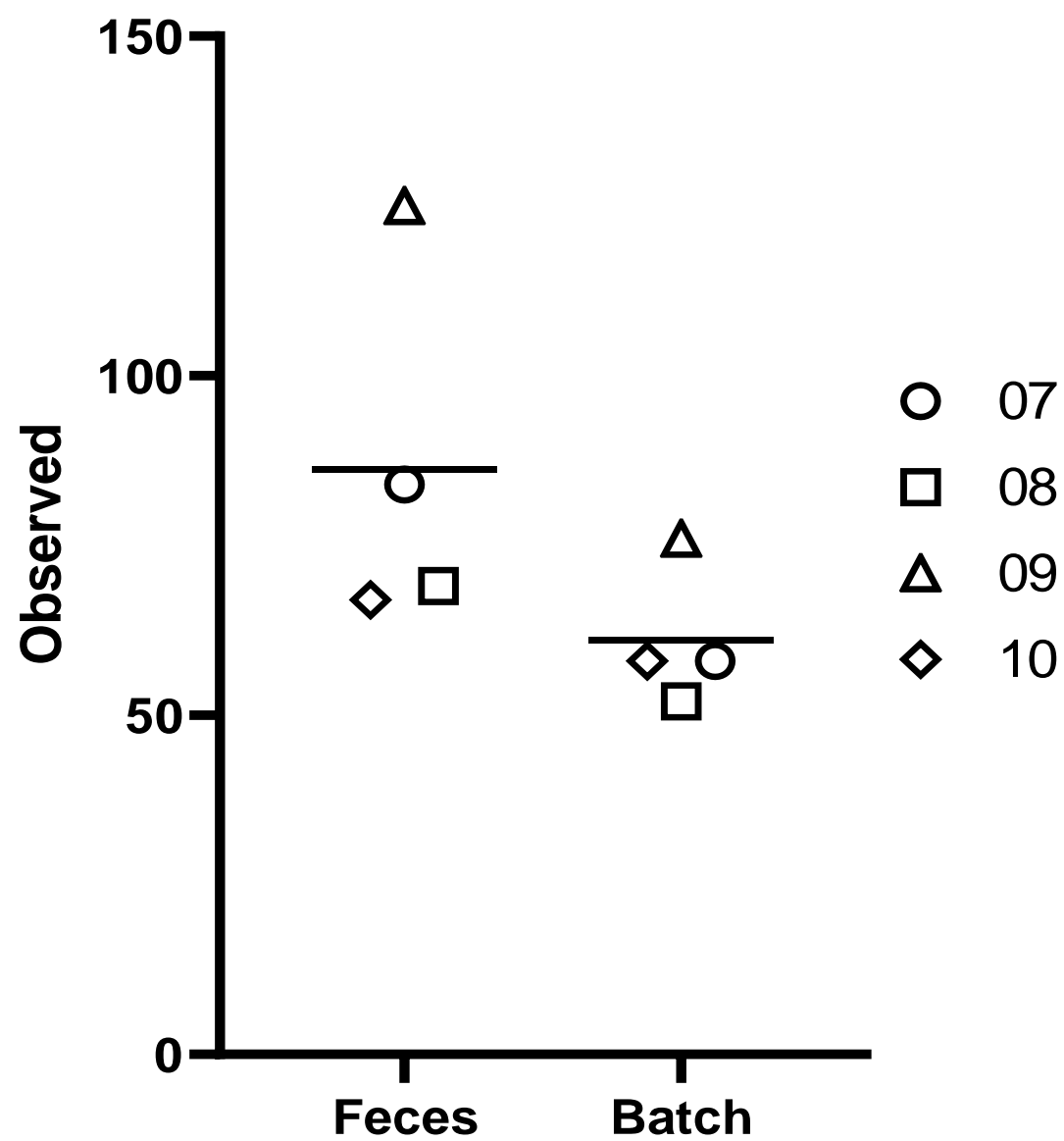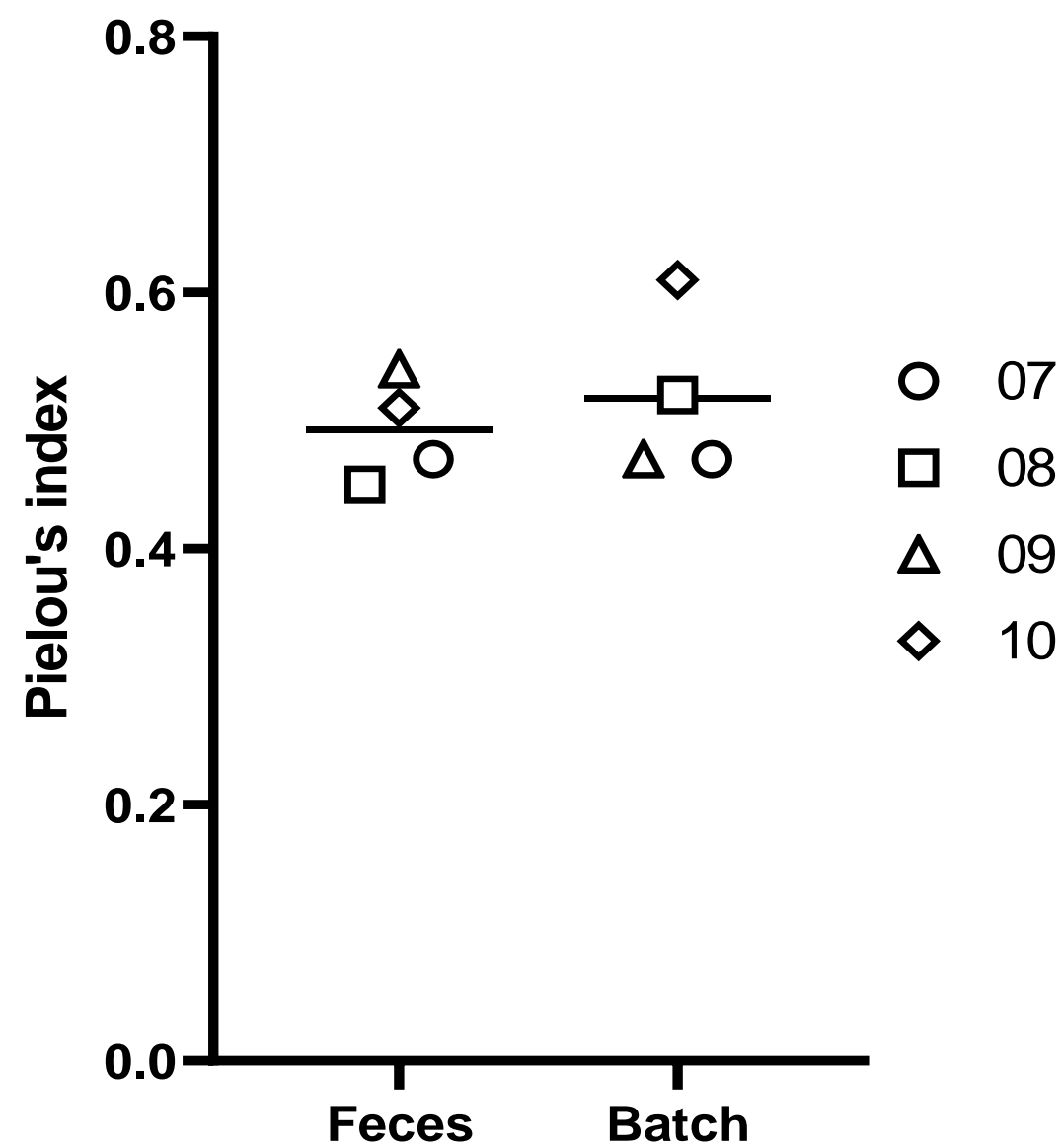

Supplement: Supplementary file 6 — Supplementary Material 6 [file 12866_2023_2915_MOESM6_ESM.pdf]
