# Supplementary material for: Validation of a batch cultivation protocol for fecal microbiota of Kenyan infants
Source: BMC Microbiol. 2023 Jul 4;23:174. doi: 10.1186/s12866-023-02915-9 (PMC10318780; doi:10.1186/s12866-023-02915-9)
Supplement: Supplementary file 8 — Supplementary Material 8 [file 12866_2023_2915_MOESM8_ESM.docx]

Additional file 1.pdf: **Supplemental Table 1**. **Composition of the cultivation medium designed to mimic the ileal chyme entering the proximal colon of Kenyan infants during weaning.**

Additional file 2.xlsx: **Supplemental Table 2. Statistical output PERMANOVA and Multivariate Dispersion Test.**

Additional file 3.pdf: **Supplemental Figure 1.** **Microbiota composition of fresh and stabilized fecal samples based on 16S rRNA gene amplicon sequencing data.** Relative abundance on genus level (“other” <1%). The numbers denote the infant.

Additional file 4.xlsx: **Supplemental Table 3.** **Initial, final and delta pH of fecal batch fermentation.**

Additional file 5.pdf: **Supplemental Figure 2.** **Beta diversity of fresh feces and after 24 h fecal batch fermentation based on 16S rRNA gene amplicon sequencing data.** Binary and weighted Jaccard distance plot is shown comparing fermentation sample to feces of the same infant (within) or of the other infants (other). Infant 01 to 06 cultivated at initial pH 7.6 (**A**). Infant 07 to 10 cultivated at initial pH 6.9 (**B**). Average ± SD is shown for “other” (n=5 for infant 01 to 06, n=3 for infant 07 to 10).

Additional file 6.pdf: **Supplemental Figure 3.** **Alpha diversity of fresh feces and after 24 h fecal batch fermentation based on 16S rRNA gene amplicon sequencing data.** Number of observed ASVs (richness) and Pielou’s index (evenness) are shown for Infant 01 to 06 cultivated at initial pH 7.6 (**A**) and Infant 07 to 10 cultivated at initial pH 6.9 (**B**). Per infant the average of technical duplicates is shown. The numbers denote the infant. An unpaired two-tailed t-test was performed between fecal and fermentation samples.

Additional file 7.xlsx: **Supplemental Table 4. Total 16S rRNA gene copies in fecal and fermentation samples based on qPCR.** The average of technical duplicates is shown for copy numbers of feces and fermentation samples. A dilution factor of 500 (1% fecal slurry containing 20% of original sample) was applied to calculate the copy numbers of inoculated fecal samples.
